# Supplementary material for: Resilience to Endoplasmic Reticulum Stress Mitigates Membrane Hyperexcitability Underlying Late Disease Onset in a Murine Model of SCA6
Source: Ann Neurol. 2025 Sep 24;99(2):502–22. doi: 10.1002/ana.78042 (PMC12894513; doi:10.1002/ana.78042)
Supplement: Supplementary file 1 — Figure S1. Purkinje neuron firing frequency and changes in ion channel transcripts/protein in SCA684Q/+ mice. Figure S2. Irregular Purkinje neuron spiking in SCA684Q/+ mice is due to changes in intrinsic membrane excitability. Figure S3. Spatial transcriptome analysis of Purkinje neurons. Figure S4. Knockout of XBP1 in Purkinje neurons has no effect on the motor phenotype, Purkinje neuron spiking regularity and firing frequency but alters other UPR pathways in SCA684Q/+ mice. Figure S5. The increase in CRAC current in 19‐month SCA684Q/+ mice is not caused by increased expression of Orai2/Stim1 subunits in cerebella. Figure S6. 17‐AAG has no effect on Purkinje neuron firing frequency in 6‐month SCA684Q/+ mice or 19‐month wild‐type mice. Table S1. Electrophysiological parameters of Purkinje neurons from 6‐month and 19‐month wild‐type and SCA684Q/+ mice. [file ANA-99-502-s002.docx]

**
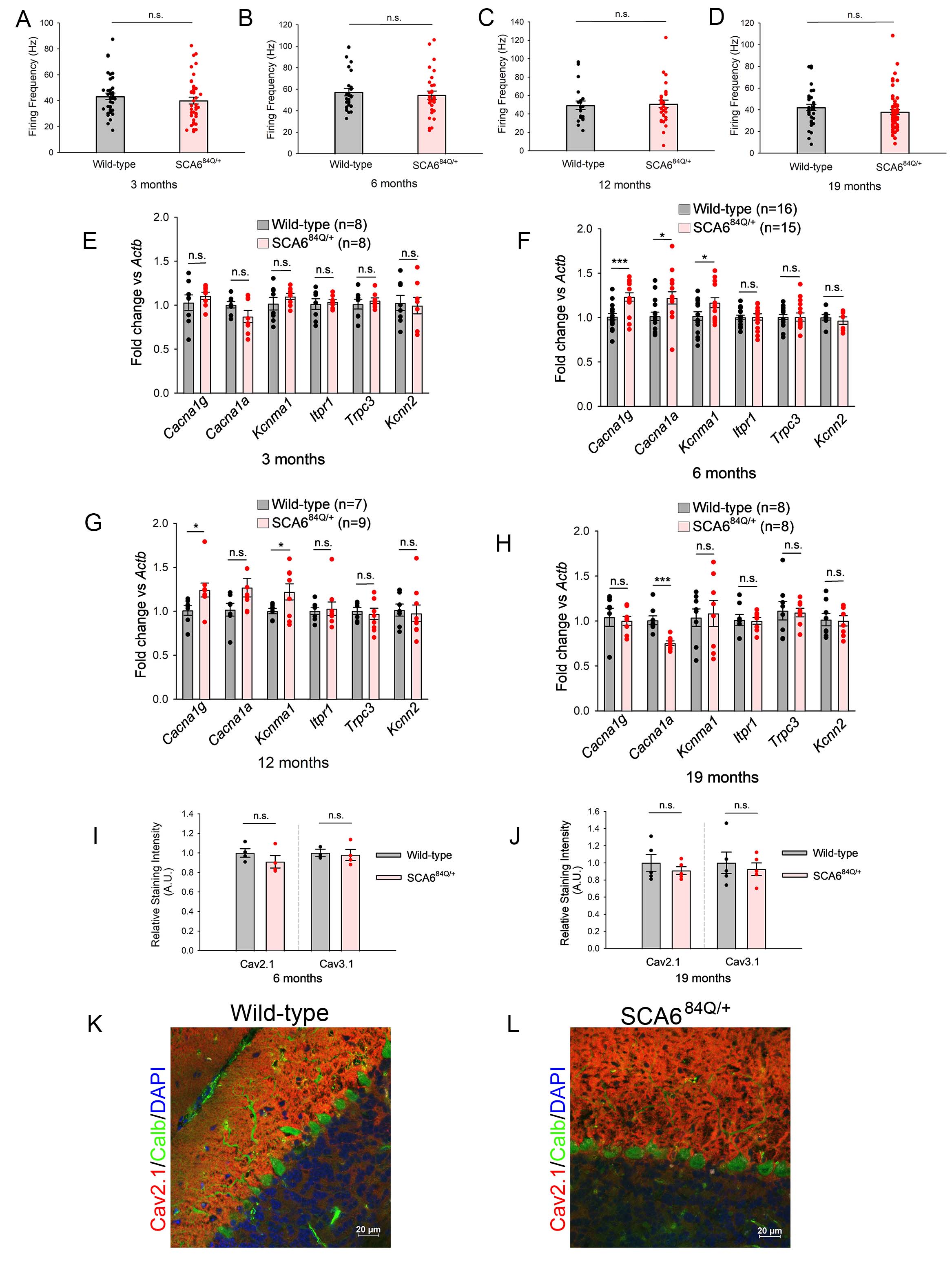
**

**Supplemental Figure 1. Purkinje neuron firing frequency and changes in ion channel transcripts/protein in SCA6^84Q/+^ mice.**

1. Purkinje neuron firing frequency is unchanged between 3-month WT (N = 3) and SCA6^84Q/+^ mice (N = 3). WT cells: n = 37, SCA6^84Q/+^ cells: n = 42. Student’s t-test, n.s.: Not significant.
2. Purkinje neuron firing frequency is unchanged between 6-month WT (N = 3) and SCA6^84Q/+^ mice (N = 3). WT cells: n=28, SCA6^84Q/+^ cells: n = 30. Student’s t-test, n.s.: Not significant.
3. Purkinje neuron firing frequency is unchanged between 12-month WT (N = 4) and SCA6^84Q/+^ mice (N = 3). WT cells: n=20, SCA6^84Q/+^ cells: n = 31. Student’s t-test, n.s.: Not significant.
4. Purkinje neuron firing frequency is unchanged between 19-month WT (N = 4) and SCA6^84Q/+^ mice (N = 11). WT cells: n=35, SCA6^84Q/+^ cells: n = 64. Student’s t-test, n.s.: Not significant.
5. Quantitative RT-PCR showing changes in the transcript levels of *Cacna1g*, *Cacna1a*, *Kcnma1*, *Itpr1*, *Trpc3*, and *Kcnn2* relative to *Actb* in 3-month WT (N = 8) and SCA6^84Q/+^ (N = 8) mice. Student’s t-test, n.s.: Not significant.
6. Quantitative RT-PCR showing changes in the transcript level of *Cacna1g*, *Cacna1a*, *Kcnma1*, *Itpr1*, *Trpc3*, and *Kcnn2* relative to *Actb* in 6-month WT (N = 16) and SCA6^84Q/+^ (N = 15) mice. Student’s t-test, *P < 0.05, ***P < 0.001, n.s.: Not significant.
7. Quantitative RT-PCR showing changes in the transcript level of *Cacna1g*, *Cacna1a*, *Kcnma1*, *Itpr1*, *Trpc3*, and *Kcnn2* relative to *Actb* in 12-month WT (N = 7) and SCA6^84Q/+^ (N = 9) mice. Student’s t-test, *P < 0.05, n.s.: Not significant.
8. Quantitative RT-PCR showing changes in the transcript level of *Cacna1g*, *Cacna1a*, *Kcnma1*, *Itpr1*, *Trpc3*, and *Kcnn2* relative to *Actb* in 19-month WT (N = 8) and SCA6^84Q/+^ (N = 8) mice. Student’s t-test, ***P < 0.001, n.s.: Not significant.
9. Quantification of immunostaining showing that protein levels of Cav2.1 and Cav3.1 are unchanged between cerebella of 6-month WT (N = 4) and SCA6^84Q/+^ (N = 4) mice. Student’s t-test, n.s.: Not significant.
10. Quantification of immunostaining showing that protein levels of Cav2.1 and Cav3.1 are unchanged between cerebella of 19-month WT (N = 5) and SCA6^84Q/+^ (N = 5) mice. Student’s t-test, n.s.: Not significant.
11. Representative image from a 19-month WT cerebellar section showing immunostaining for Cav2.1 (red), Calbindin (green) and DAPI (blue).
12. Representative image from a 19-month SCA6^84Q/+^ cerebellar section showing immunostaining for Cav2.1 (red), Calbindin (green) and DAPI (blue).

**
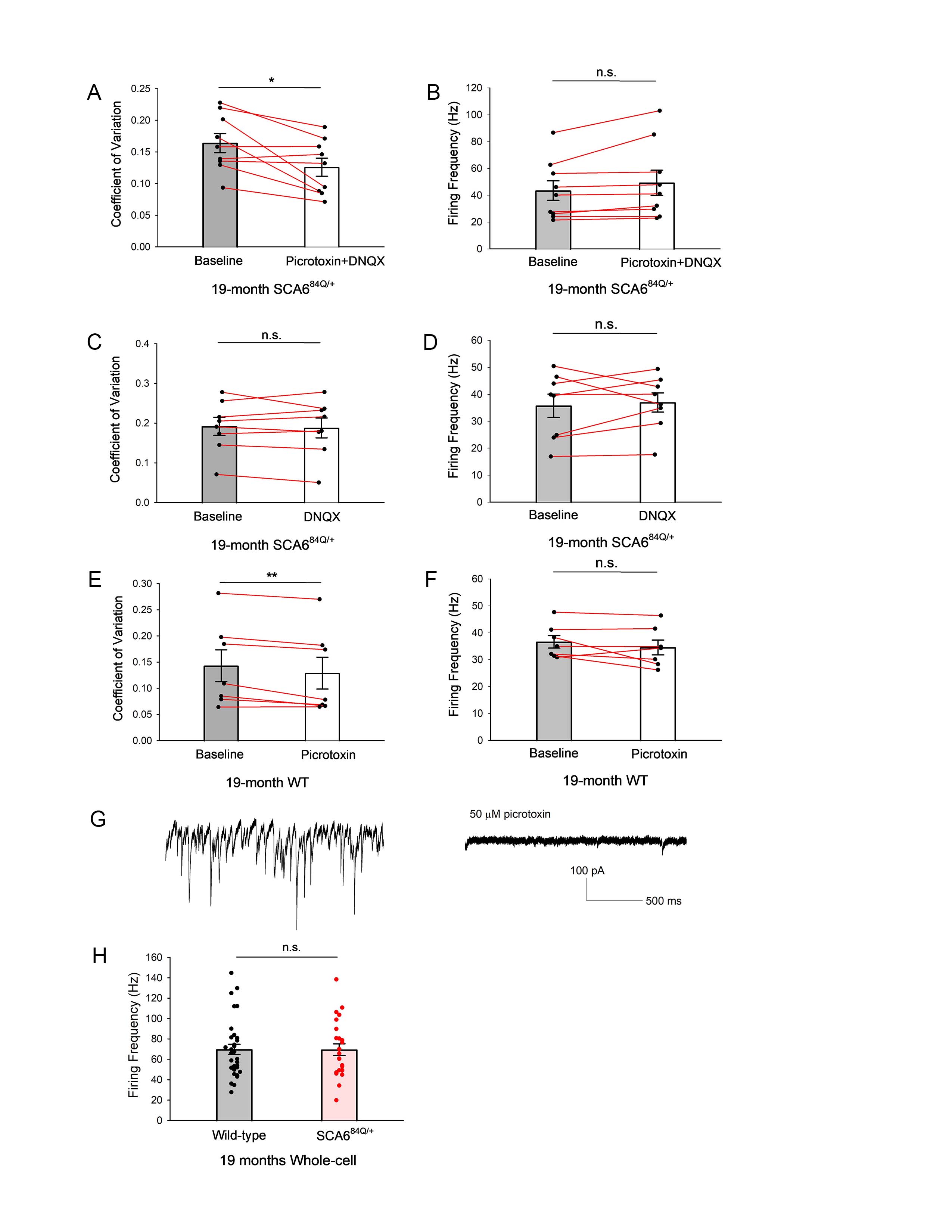
**

**Supplemental Figure 2. Irregular Purkinje neuron spiking in SCA6^84Q/+^ mice is due to changes in intrinsic membrane excitability**

1. A combination of picrotoxin and DNQX improves Purkinje neuron spiking irregularity in 19-month SCA6^84Q/+^ mice (N = 3). n = 9 cells. Paired t-test, *P < 0.05.
2. A combination of picrotoxin and DNQX has no effect on Purkinje neuron firing frequency in 19-month SCA6^84Q/+^ mice (N = 3). n = 9 cells. Paired t-test, n.s.: Not significant.
3. DNQX alone has no effect on Purkinje neuron spiking regularity in 19-month SCA6^84Q/+^ mice (N = 2). n = 8 cells. Paired t-test, n.s.: Not significant.
4. DNQX alone has no effect on Purkinje neuron firing frequency in 19-month SCA6^84Q/+^ mice (N = 2). n = 8 cells. Paired t-test, n.s.: Not significant.
5. Picrotoxin improves Purkinje neuron spiking irregularity in 19-month WT mice (N = 2). n = 7 cells. Paired t-test, *P < 0.05.
6. Picrotoxin has no effect on Purkinje neuron firing frequency in 19-month WT mice (N = 2). n = 7 cells. Paired t-test, n.s.: Not significant.
7. Representative traces of IPSCs before (left) and after (right) picrotoxin perfusion.
8. Purkinje neuron firing frequency in the whole-cell patch clamp configuration is comparable between 19-month WT (N = 4) and SCA6^84Q/+^ (N = 9) mice. WT cells: n = 33, SCA6^84Q/+^ cells: n = 24. Student’s t-test, n.s.: Not significant.


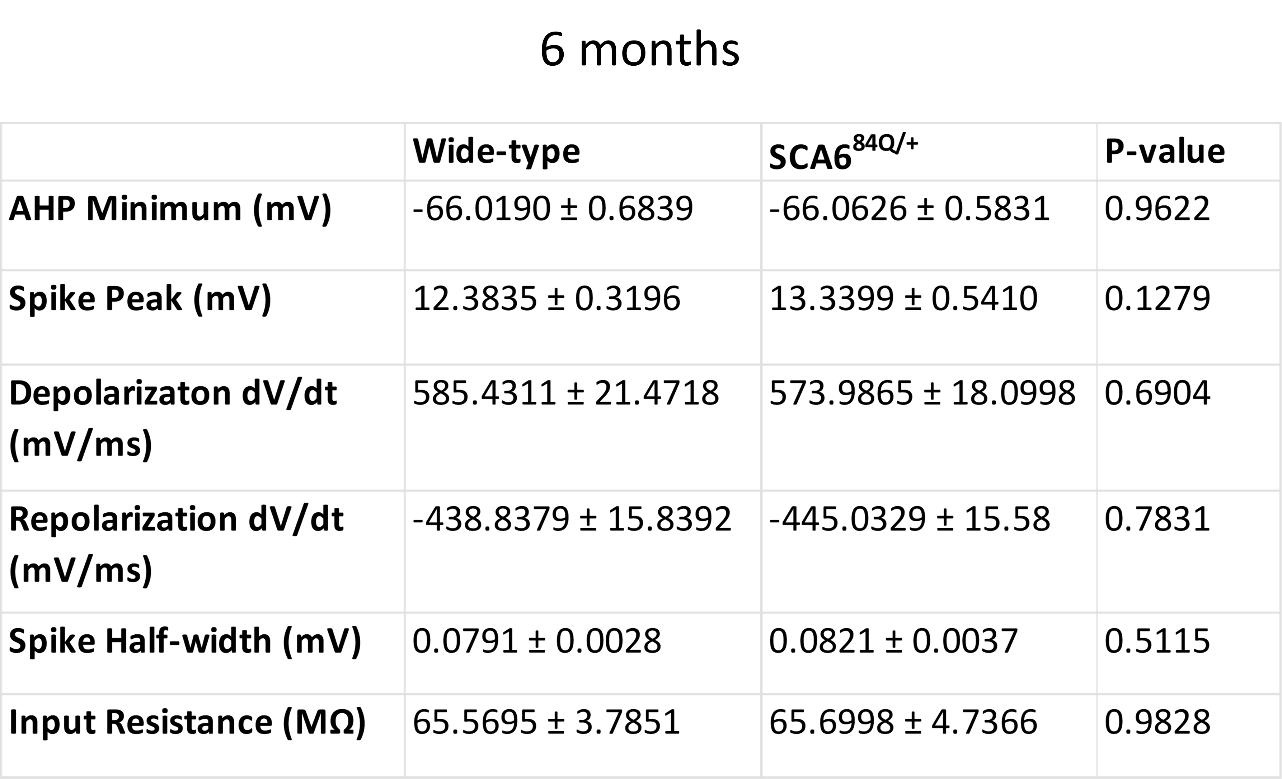

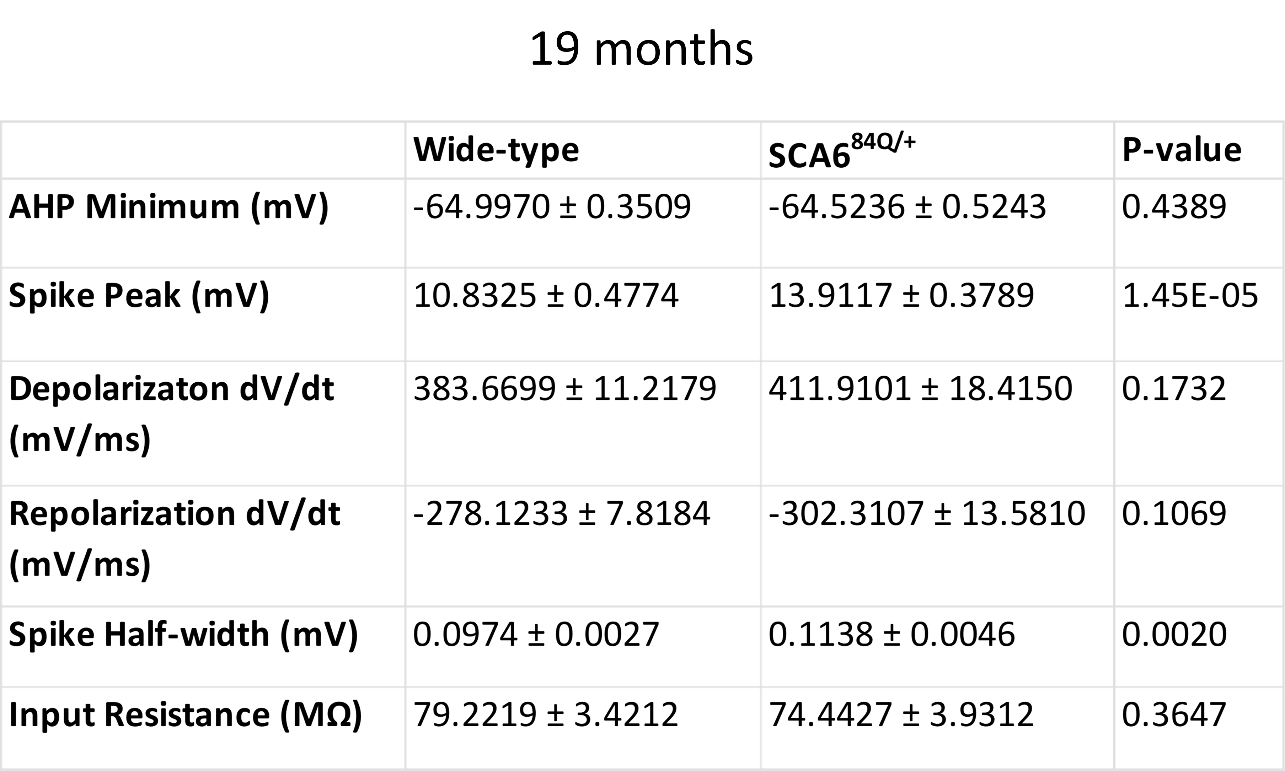


**Supplemental Table 1. Electrophysiological parameters of Purkinje neurons from 6-month and 19-month wild-type and SCA6^84Q/+^ mice**.

Data are presented as mean ± standard error of the mean. Two-tailed Student’s t-test.

**
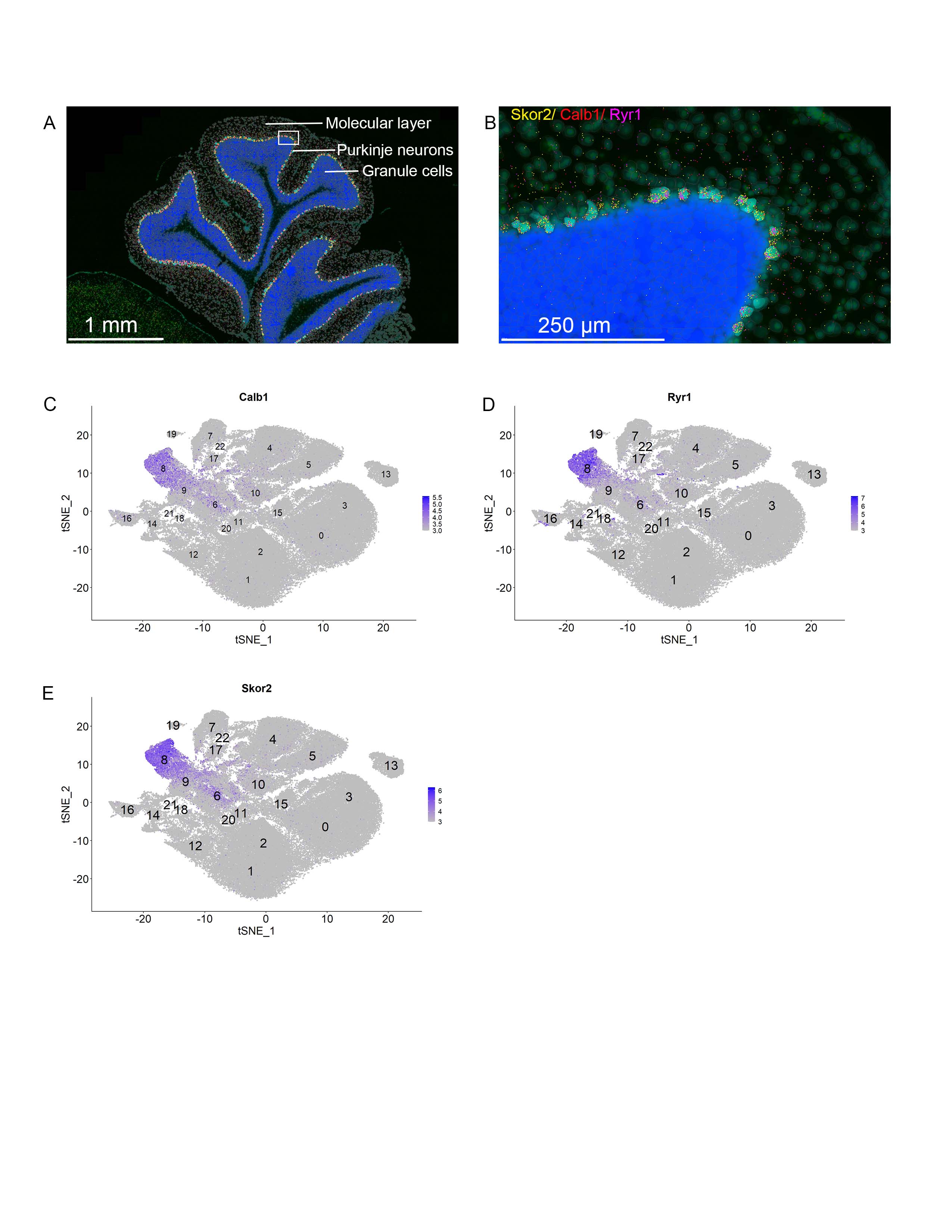
**

**Supplemental Figure 3. Spatial transcriptome analysis of Purkinje neurons**

1. Trascripts of Calb1 (yellow), Skor2 (red), and Ryr1 (pink) mark the somata of Purkinje neurons using multiplexed error-robust fluorescent in situ hybridization (MERFISH).
2. Expanded view of the rectangular area in A), also showing cell nuclei stained with DAPI (blue) and RNA poly (A) tail staining in green.
3. Cell cluster 8 is enriched for *Calb1* transcripts, *Ryr1* (D) and *Skor2* (E) transcripts, indicating that this cell cluster represents Purkinje neurons.

**
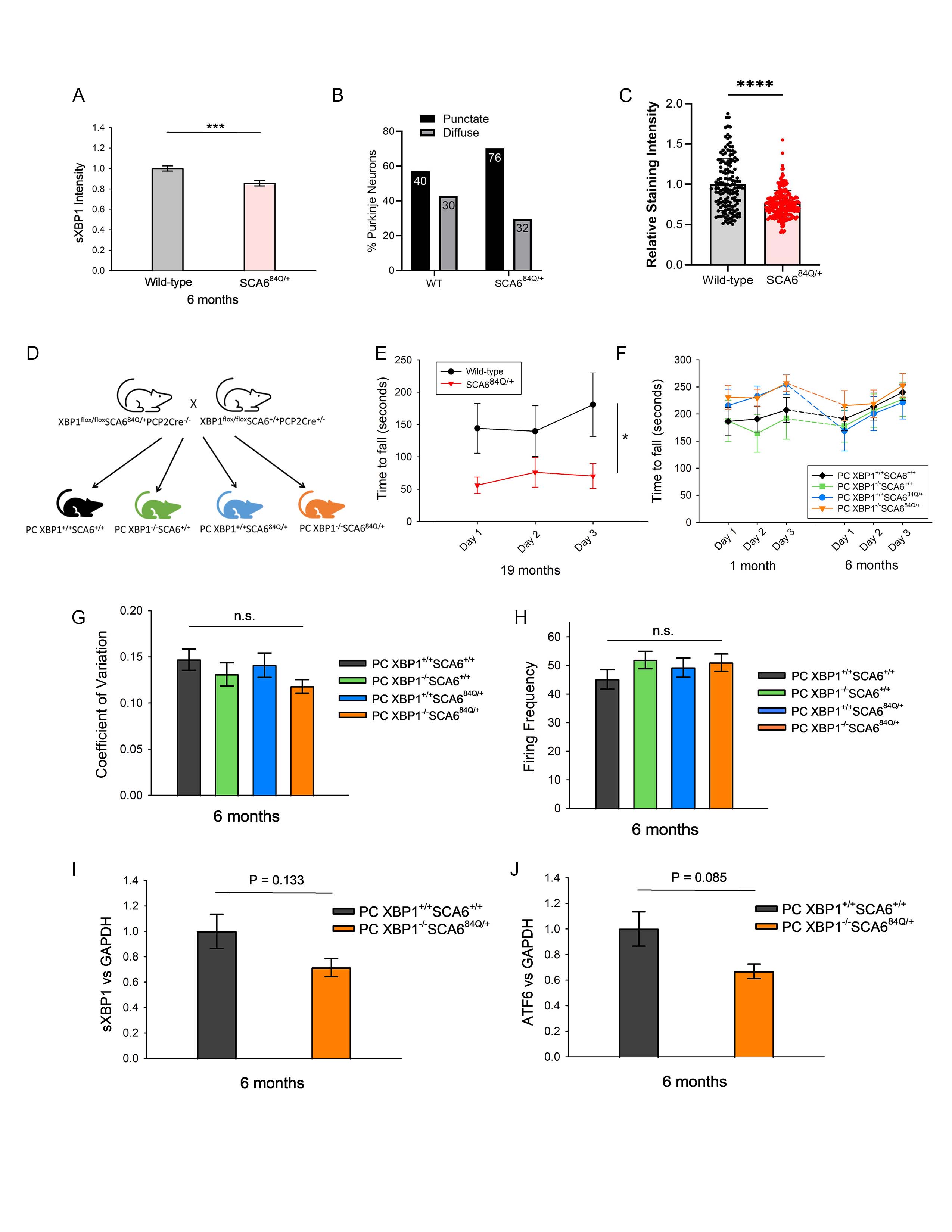
**

**Supplemental Figure 4. Knockout of XBP1 in Purkinje neurons has no effect on the motor phenotype, Purkinje neuron spiking regularity and firing frequency but alters other UPR pathways in SCA6^84Q/+^ mice.**

1. Quantification of sXBP1 intensity in nuclei of 6-month WT (n = 39 cells) and SCA6^84Q/+^ (n = 65 cells) Purkinje neurons. The nuclear sXBP1 intensity is significantly reduced in SCA6^84Q/+^ Purkinje neurons. N = 5 mice of each genotype. Student’s t-test, ***P < 0.001.
2. Counts of Purkinje neurons with a punctate or diffuse pattern of sXBP1 staining revealed a greater proportion of SCA6^84Q/+^ mice with a punctate pattern of sXBP1 compared to WT littermate controls, although this was not statistically significant. Fisher’s exact test, P = 0.078. Numbers of Purkinje neurons with each pattern shown within the bars. N = 5 mice of each genotype.
3. Quantification of ATF6 intensity in 6-month WT (N = 4 mice, n = 151 cells) and SCA6^84Q/+^ (N = 5 mice, n = 229 cells) Purkinje neurons. ATF6 staining intensity is significantly reduced in SCA6^84Q/+^ Purkinje neurons. Student’s t-test, ****P < 0.0001.
4. Mouse breeding scheme showing the generation of PC XBP1^+/+^SCA6^+/+^ (XBP1^flox/flox^SCA6^+/+^ PCP2Cre^-/-^), PC XBP1^-/-^SCA6^+/+^ (XBP1^flox/flox^SCA6^+/+^ PCP2Cre^+/-^), PC XBP1^+/+^SCA6^84Q/+^ (XBP1^flox/flox^SCA6^84Q /+^ PCP2Cre^-/-^), and PC XBP1^-/-^SCA6^84Q/+^ (XBP1^flox/flox^SCA6^84Q /+^ PCP2Cre^+/-^) mice.
5. 19-month SCA6^84Q/+^ mice (N = 11) perform significantly worse on the rotarod compared to age-matched WT mice (N = 7). Student’s t-test, *P < 0.05.
6. Motor performance on the rotarod is comparable among PC XBP1^+/+^SCA6^+/+^ mice (N = 15), PC XBP1^-/-^SCA6^+/+^ mice (N = 9), PC XBP1^+/+^SCA6^84Q/+^ mice (N = 7), and PC XBP1^-/-^SCA6^84Q/+^ mice (N = 12) at both 1-month and 6-months of age. Two-way repeated measures ANOVA.
7. Purkinje neuron spiking regularity, indicated by the coefficient of variation of the interspike interval, is comparable among PC XBP1^+/+^SCA6^+/+^ mice (N = 6 mice, n = 33 cells), PC XBP1^-/-^SCA6^+/+^ mice (N = 3 mice, n = 30 cells), PC XBP1^+/+^SCA6^84Q/+^ mice (N = 3 mice, n = 34 cells), and PC XBP1^-/-^SCA6^84Q/+^ mice (N = 4 mice, n = 34 cells) at 6 months of age. One way ANOVA, n.s.: Not significant.
8. Purkinje neuron firing frequency is unchanged among PC XBP1^+/+^SCA6^+/+^ mice (N = 6 mice, n = 33 cells), PC XBP1^-/-^SCA6^+/+^ mice (N = 3 mice, n = 30 cells), PC XBP1^+/+^SCA6^84Q/+^ mice (N = 3 mice, n = 34 cells), and PC XBP1^-/-^SCA6^84Q/+^ mice (N = 3 mice, n = 30 cells) at 6 months of age. One way ANOVA, n.s.: Not significant.
9. Quantification of cerebellar sXBP1 levels in female PC XBP1^-/-^SCA6^84Q/+^ mice (N = 3) compared to female PC XBP1^+/+^SCA6^+/+^ mice (N = 3). Cerebellar sXBP1 levels are reduced in female PC XBP1^-/-^SCA6^84Q/+^ mice but this reduction is not statistically significant. Student’s t-test.
10. Quantification of cerebellar ATF6 levels in female PC XBP1^-/-^SCA6^84Q/+^ mice (N = 3) compared to female PC XBP1^+/+^SCA6^+/+^ mice (N = 3). Cerebellar ATF6 levels are reduced in female PC XBP1^-/-^SCA6^84Q/+^ mice but this reduction is not statistically significant. Student’s t-test.

**
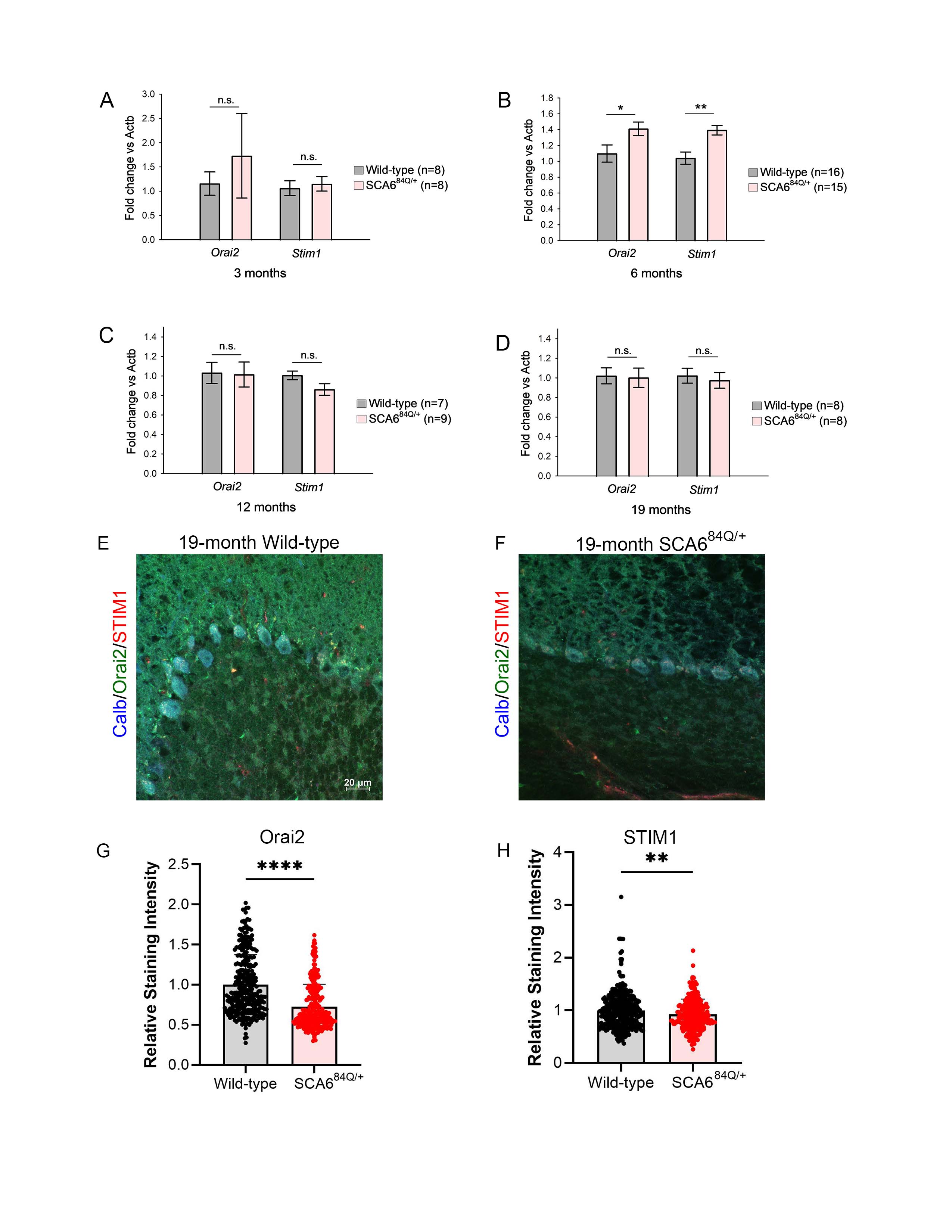
Supplemental Figure 5. The increase in CRAC current in 19-month SCA6^84Q/+^ mice is not caused by increased expression of Orai2/Stim1 subunits in cerebella.**

1. Quantitative RT-PCR shows no changes in *Orai2* or *Stim1* transcript levels relative to *Actb* in cerebella of 3-month SCA6^84Q/+^ mice (N = 8) compared to WT mice (N = 8). Student’s t-test, n.s.: Not significant.
2. Quantitative RT-PCR shows a small increase of both *Orai2* and *Stim1* transcripts relative to *Actb* in the cerebella of 6-month SCA6^84Q/+^ mice (N = 15) compared to WT mice (N = 16). Student’s t-test, *P < 0.05, **P < 0.01.
3. Quantitative RT-PCR shows no changes in *Orai2* or *Stim1* transcript levels relative to *Actb* in cerebella of 12-month SCA6^84Q/+^ mice (N = 9) compared to WT mice (N = 7). Student’s t-test, n.s.: Not significant.
4. Quantitative RT-PCR shows no changes in *Orai2* or *Stim1* transcript levels relative to *Actb* in cerebella of 19-month SCA6^84Q/+^ mice (N = 8) compared to WT mice (N = 8). Student’s t-test, n.s.: Not significant.
5. Representative immunostaining images showing Orai2 (green), STIM1 (red), and Calbindin (blue) in 19-month WT mice.
6. Representative immunostaining images showing Orai2 (green), STIM1 (red), and Calbindin (blue) in 19-month SCA6^84Q/+^ mice.
7. Quantification of Orai2 intensity in 19-month WT (N = 5 mice, n = 311 cells) and SCA6^84Q/+^ (N = 5 mice, 258 cells) Purkinje neurons. Orai2 staining intensity is significantly lower in SCA6^84Q/+^ Purkinje neurons. Student’s t-test, ****P < 0.0001.
8. Quantification of STIM1 intensity in 19-month WT (N = 5 mice, n = 202 cells) and SCA6^84Q/+^ (N = 5 mice, n = 258 cells) Purkinje neurons. STIM1 staining intensity is significantly lower in SCA6^84Q/+^ Purkinje neurons. Student’s t-test, **P < 0.01.

**
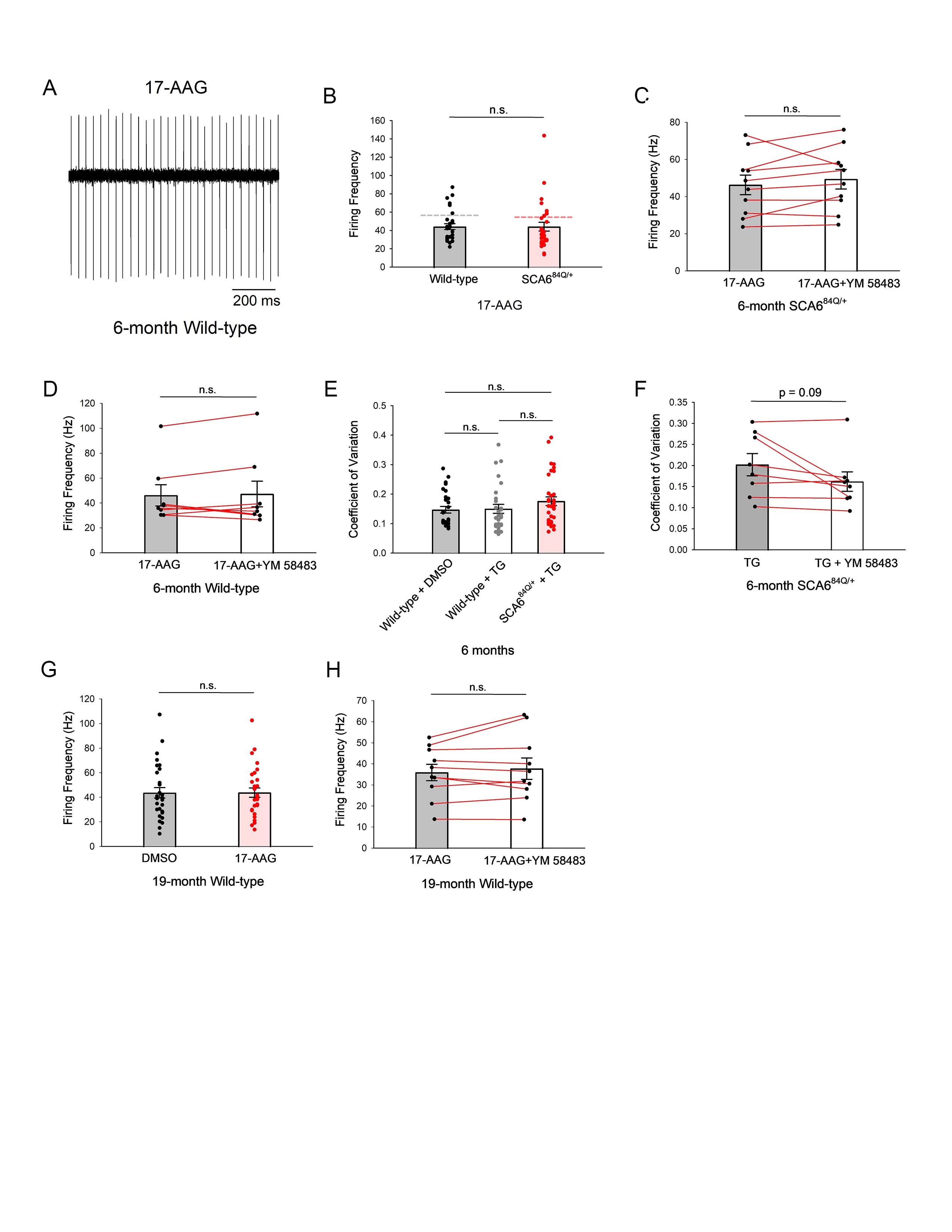
**

**Supplemental Figure 6. 17-AAG has no effect on Purkinje neuron firing frequency in 6-month SCA6^84Q/+^ mice or 19-month wild-type mice.**

1. Representative trace of spontaneous Purkinje neuron spiking in 17-AAG pre-incubated cerebellar slices from 6-month WT mice.
2. 17-AAG does not change Purkinje neuron firing frequency in 6-month WT (N = 3) or SCA6^84Q/+^ (N = 5) mice. Dashed lines represent the mean baseline firing frequency from Figure S1B. WT cells: n = 29, SCA6^84Q/+^ cells: n = 30. Student’s t-test, n.s.: Not significant.
3. YM 58483 does not alter firing frequency in 17-AAG-pretreated Purkinje neurons in 6-month SCA6^84Q/+^ mice (N = 2). n = 10 cells. Paired t-test, n.s.: Not significant.
4. YM 58483 does not alter firing frequency in 17-AAG-pretreated Purkinje neurons in 6-month WT mice (N = 2). n = 8 cells. Paired t-test, n.s.: Not significant.
5. ER calcium depletion with thapsigargin (TG, 1 μM) fails to induce spiking irregularity in 6-month WT and SCA6^84Q/+^ Purkinje neurons. (WT DMSO; n = 28, N = 3 mice, WT thapsigargin; n = 28, N = 3 mice, SCA6^84Q/+^; n = 34, N = 4 mice).
6. YM 58483 fails to significantly improve firing regularity in SCA6^84Q/+^ Purkinje neurons treated with thapsigargin. Paired t-test, n = 8 cells, N = 2 mice.
7. 17-AAG has no effect on Purkinje neuron firing frequency in 19-month WT mice. DMSO-treated mice: N = 2, DMSO-treated cells: n = 29; 17-AAG-treated mice: N = 3, 17-AAG-treated cells: n = 29. Student’s t-test, n.s.: Not significant.
8. YM 58483 has no effect on Purkinje neuron firing frequency in 17-AAG-pretreated 19-month WT cerebellar slices (N = 2 mice). n = 10 cells. Paired t-test, n.s.: Not significant.
